# Supplementary figures and images for: Extensive ribosome and RF2 rearrangements during translation termination
Source: eLife. 2019 Sep 12;8:e46850. doi: 10.7554/eLife.46850 (PMC6742477; doi:10.7554/eLife.46850)

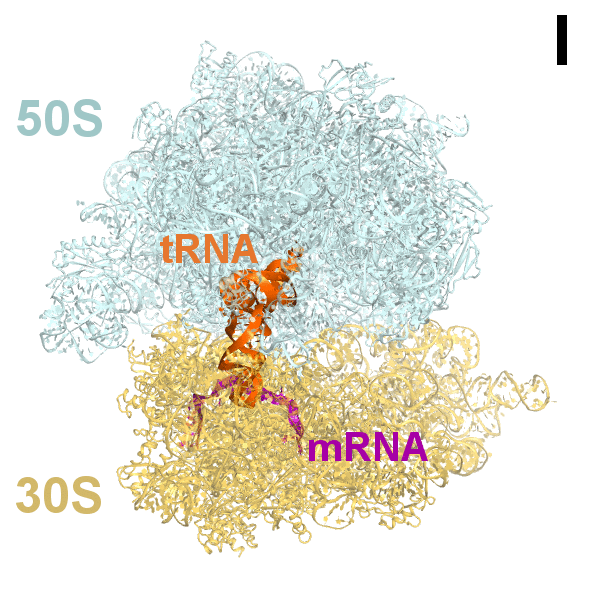

Supplement: Supplementary file 1 [file elife-46850-video1.gif]
